# Supplementary material for: Identification of Quantitative Trait Loci (QTL) for Sucrose and Protein Content in Soybean Seed
Source: Plants (Basel). 2024 Feb 27;13(5):650. doi: 10.3390/plants13050650 (PMC10934403; doi:10.3390/plants13050650)
Supplement: Supplementary file 1 [file plants-13-00650-s001.zip › Supplementary Figures S1-S5.pdf]

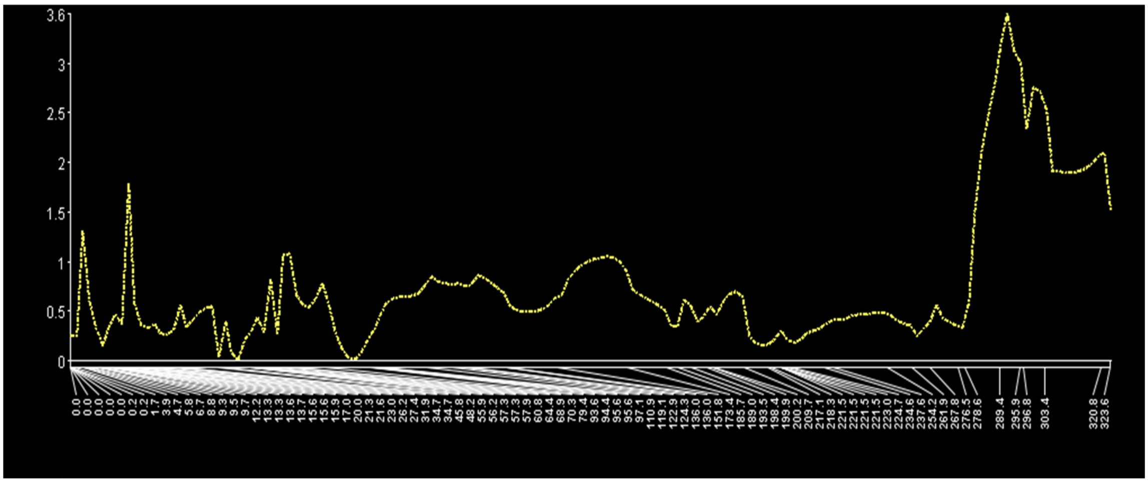

Figure S1. QTL for total seed protein on Chr. 11 shows the QTL curve for total seed protein. As shown, the QTL's peak with an LOD score of approximately 3.6. In addition, the additive effects are positive for protein content.

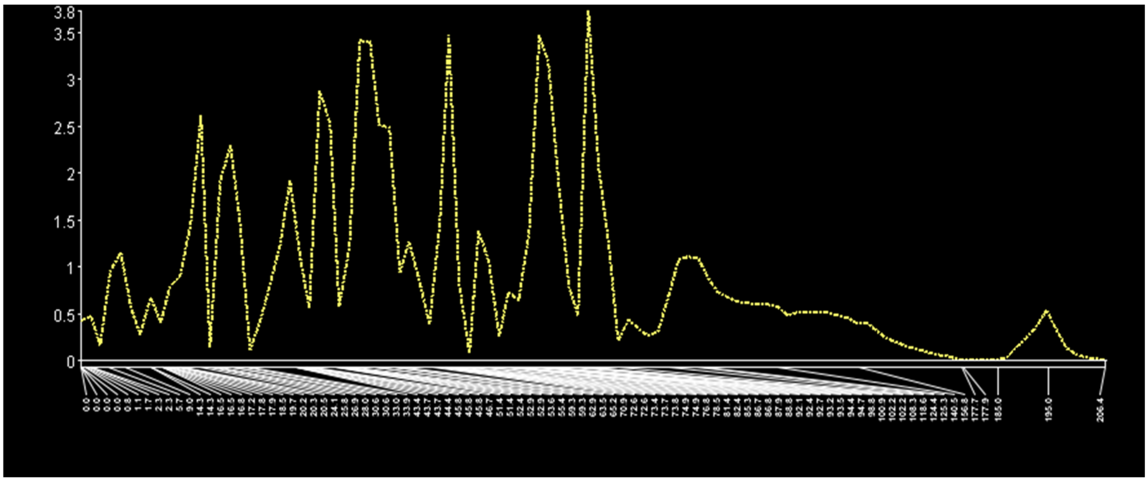

Figure S2. QTL for total seed protein on Chr. 20. As shown in (A) the QTL curve for total seed protein. As shown, QTL's peak with an LOD score of approximately 3.8.

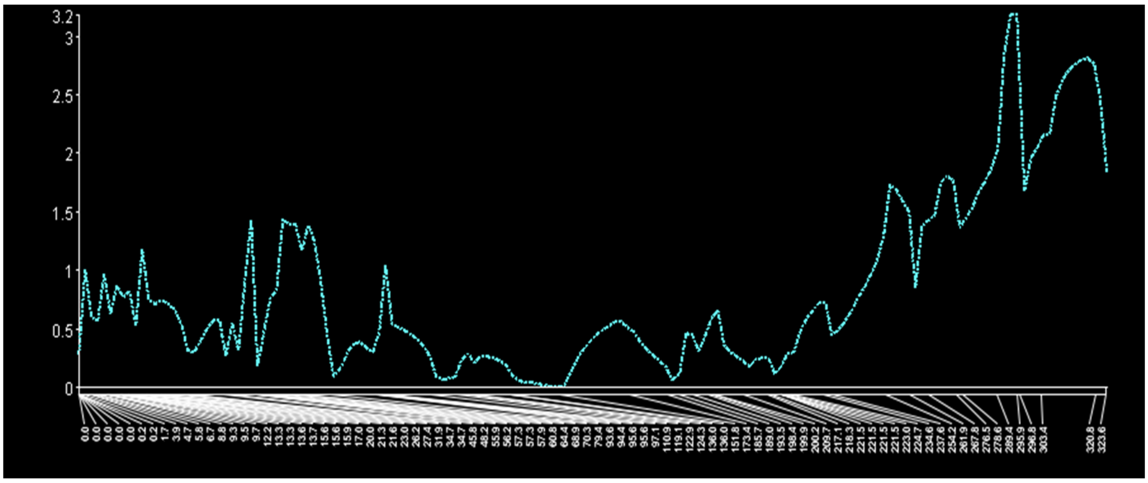

Figure S3. QTL for total sucrose on Chr. 11. A represents the QTL's position in the QTL, and B represents the additive effect on the trait. The figure shows the QTL curve for sucrose. The QTL peaks with an LOD score of approximately 3.2.

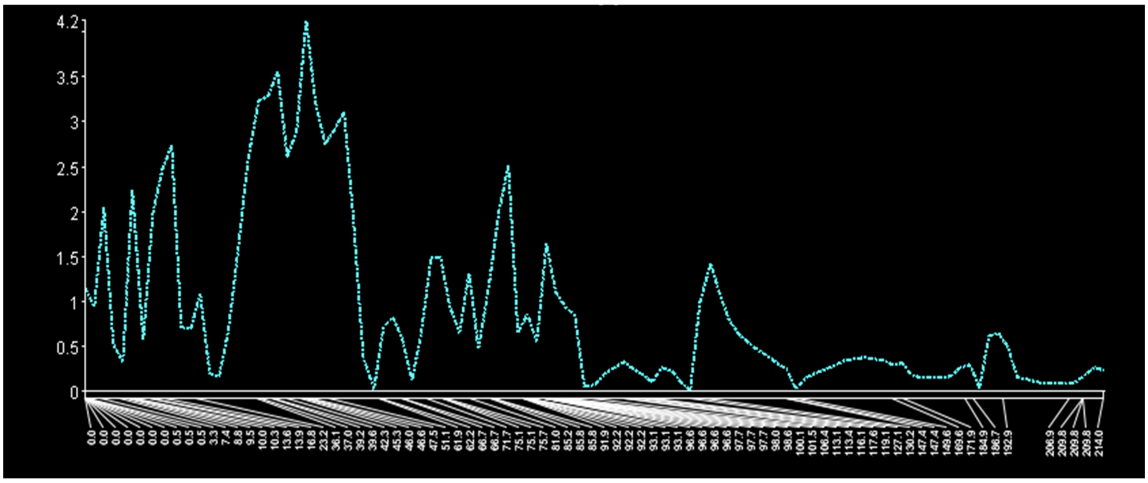

Figure S4. QTL for total sucrose on Chr. 14. The figure shows the QTL curve for sucrose. The QTL's peak with an LOD score of approximately 4.2 is shown.

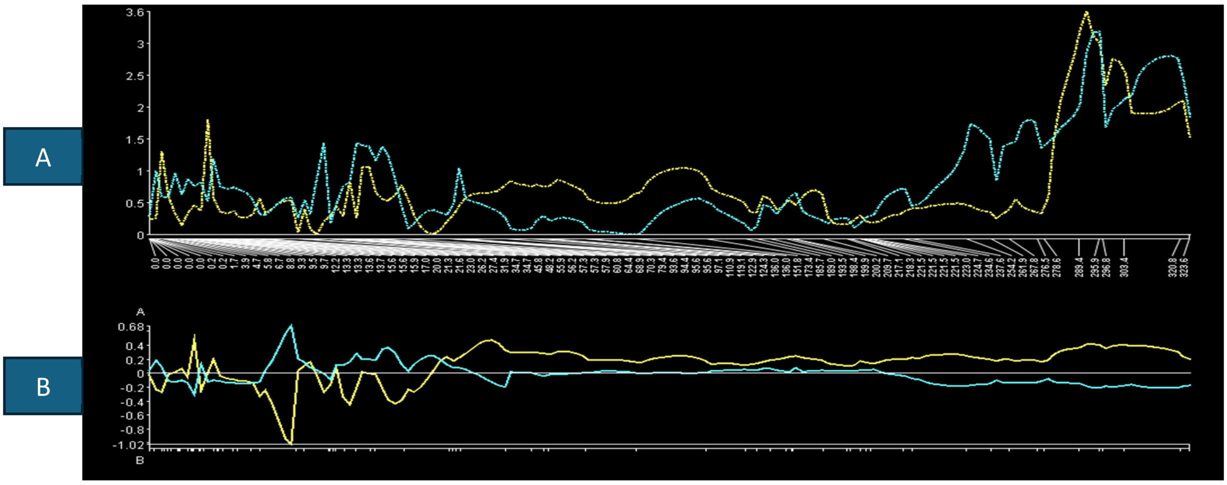

Figure S5. QTL for total protein and sucrose on Chr. 11. QTL curves for total seed protein (yellow) and sucrose (blue) content on the same plot. Figure (A) represents the location of QTL in the chromosome, and figure (B) represents the interaction between sucrose and protein. As shown in (A), the LOD scores were 3.6. and 3.4 for protein and sucrose, respectively. Figure B revealed a negative correlation between protein and sucrose contents.
